# Exiled lives on the stage: Support networks and programs for artists at risk from Turkey in Germany

**DOI:** 10.12688/openreseurope.15726.1

**Published:** 2023-07-06

**Authors:** Pieter Verstraete

**Affiliations:** 1 Institut für Theaterwissenschaft, Free University of Berlin, Berlin, 12165, Germany; 2 Arts, Culture and Media, University of Groningen, Groningen, 9712 GC, The Netherlands

**Keywords:** policy, cultural politics, diaspora, Turkey, Berlin, performing arts, risk, cultural entrepreneurship

## Abstract

This article analyses the support and self-care strategies of artists from Turkey who have left their country from considerable risk regarding their country’s political and economic instability since 2013 and have relocated to Germany. It maps the support networks, programs and institutions as well as consider questions of sustainability and risk (self-) assessment. The study is based on interviews with Turkish and Kurdish artists in Germany and an analysis of the activities by the Maxim Gorki Theater, Apartment Projekt, bi’bak, Hafiza Merkezi Berlin, as well as support systems like artistic research fellowships, art residencies, artist networks and supportive theatres. The guiding questions of this study examine the longer-term orientation of the support of artists at risk.

The findings show three deficiencies. Despite the variety of available support systems, artists who left Turkey experience difficulties integrating in the artistic labour market. The output-oriented, meritocratic basis on which programs select candidates often fail to help artists in a holistic way. The intergenerational disparity in the migrant communities create the infrastructures, solidarity discourses and networks for newly arriving artists, but also creates ideological tensions which limits inter-communitarian solidarity. Specific self-organized programs extend the solidarity to artists from other affected regions, which limits chances to support artists at risk from Turkey. There are generational disadvantages of newcomers in an overburdened, professionalized independent art scene that has struggled to break free from the social work and socio-cultural stigmas. The latter masks artists with a non-German background from positive discrimination initiatives.

The study proposes improvement in support systems through a coordinated effort, encourages to relieve programs from a need for identity representation as an expectation or requirement, and advises a shift in support systems from output orientation towards enhancement of individuals through self-care, for greater autonomy and self-development in their art practices and new life.

## Plain language summary

This analysis looks critically at the existing opportunities for artists at risk from Turkey in Germany, focusing on the independent art scene in Berlin. It offers an overview of the most relevant support networks, programs and institutions until the summer of 2022. It also determines common deficiencies and possible solutions as recommendations to improve the existing support mechanisms towards more sustainability. This article aims to help, thereby, circulating the information around existing programs and residencies, as well as to help newly arriving artists at risk in harnessing self-care.

## Introduction

Since the decline of civic space in Turkey ^1^ starting from the period after the Gezi protests in the summer of 2013 and, more progressively, after the July 15 coup attempt in 2016, there has been a silent but constant exodus of artists from Turkey emigrating to Germany. Reasons for their relocation are: socially, the curbing of freedoms like the freedom of association, assembly, and expression; politically, a steady decline of democracy and civil society towards authoritarianism, the constant erosion of reproductive rights, the withdrawal of the Istanbul Convention in 2021 (protecting women against violence), increasing hate crimes against other, subaltern identities like the Kurdish and LGBTQ+ identities; and economically, the macro-financial instability with occasional drops of the Turkish lira with a record low of nearly TRY 15 to USD 1 in December 2021 ( *DW*, 13 Dec. 2021), affecting artistic labour as well as the whole creative industry.

Turkey’s democratic backsliding is caused by a domestic agenda of the government relying on repression based on short-term political calculations. This trend has been set in motion since the heavy-weight crackdown on protestors during the Gezi Park uprising between 28 May and mid-July 2013, which constituted a major challenge in the 19 years of rule of Recep Tayyip Erdoğan’s conservative Justice and Development Party ( *Adalet ve Kalkınma Partisi*, AKP). The protestors’ demands for a more participatory democracy by occupying public spaces were met with increased repression.

In the midst of ongoing harassment and intimidations, a referendum was held on 16 April 2017, after which Turkey’s constitutional Republic was restructured with constitutional amendments on the basis of a slim majority from a parliamentary system to a presidential representative democracy, concentrating all executive powers in the president. The latter has expanded presidential veto and the power to use presidential decrees which cannot be challenged before the Council of State, the highest administrative court ( Adar & Seufert, 2021: p. 9). These decrees were used extensively during the two-year state of emergency (2016–2018) following the attempted coup, which according to figures provided by CIVICUS on 30 October 2018, led to the shutdown of more than 1400 associations with parliamentary decrees, 15,1967 layoffs, 13,6995 detainments with 7,7524 people arrested. 5822 academics lost their jobs, 15 universities were closed and whole departments were purged ( Akpinar, 2019: 62), like the Department of Theater at Ankara University that lost almost of all its faculty staff, and 319 journalists were arrested in Turkey on grounds of alleged links to the failed coup. The US-based Islamic cleric and former ally of Erdoğan, Fethullah Gülen has been blamed for organizing the coup. Accordingly, the official state narrative around the Gezi uprising has changed numerous times and blamed ‘foreign actors’ including the philanthropist George Soros as well as police officers who belonged to Gülen’s so-called FETÖ-movement.

The new presidential governance system has further led to the corrosion of judicial independence and the rule of law. Symbolical blows were the continued detainment of the former People's Democratic Party ( *Halkların Demokratik Partisi*, HDP) co-chair Selahattin Demirtaş on unsubstantiated terrorism-related charges since November 2016, in spite of two binding rulings in favour of his release by the European Court of Human Rights; as well as the latest trial of 16 citizens who allegedly organized and financed the Gezi protests, including Turkish businessman, human rights defender and philanthropist Osman Kavala who has been in detention since his arrest (without indictment) in 2017. On 25 April 2022, Kavala was sentenced to life in prison without parole on charges of attempting to overthrow the government, and seven co-defendants were sentenced to 18 years on charges of aiding and abetting. In both cases, Human Rights Watch reported gross miscarriages of justice ( *HRW*, 7 June 2022; *HRW*, 26 April 2022).

Within this space of growing societal and economic pressures, intimidations, (self)censorship practices, a growing number of artists from Turkey who identify with a ‘new wave’ have relocated to Germany. ^2^ Their exodus is part of a growing internationalization and professionalization of artist networks, which is benefiting from global processes of artist exchange and a general interest of solidarity in Europe. Germany is a common host country to displaced artists who receive special funds, artist residencies, touring opportunities, which offer short-term solutions but are limited in time. Most artists are expected to return to their home countries after a few years or integrate into the highly competitive German cultural market, which has its own institutional inequalities.

Moreover, during the Merkel era, the degree of transactionalism in EU/German-Turkey relations has been considerably increased, which sometimes complicates the adversary language in the narratives around Turkey. For instance, the ‘EU-Turkey refugee deal’ that was signed in 2016 as a ‘statement of cooperation’ between the EU and the Turkish Government in order to externalize European migration management to third countries, meant that Turkey is increasingly regarded as a safe third country despite its human rights violations. ^3^ This also had significant impact on the financial support mechanisms for those who have left Turkey due to political prosecution and cannot simply return. New displaced peoples, like the Ukrainians, are now prioritized. Yet those who have fled from Turkey or other countries earlier cannot be expected to be integrated into the German job market so quickly, given the historical and linguistic disadvantages most experience ( Telli-Aydemir & Diner, 2021). ^4^

Notwithstanding the disadvantages and short-term capabilities, Germany, and particularly its capital Berlin, has an attractive pool factor as it is a country shaped by migrations with a significant historical connection to Turkey starting with the bilateral guest worker recruitment agreement, mostly for manufacturing, in 1961. Germany-Turkey military, industrial and trade relations date, however, already from the 18 ^th^ century starting from the declaration of peace and friendship between the Kingdom of Prussia and the Ottoman Empire in 1790, which helped migration of German artists and officials to mainly Istanbul up to the First World War. Since 1933, Ankara and Istanbul received a second wave of immigrants from Germany, especially academics and experts fleeing Nazi Germany, who were welcomed in Turkish universities and ministries to help build the newly established nation.

Seen from this historical background, there is strategic purpose to harness the intersocietal relations between Germany and Turkey, and support artists and other cultural actors who struggled for civil liberties and human rights that are currently under threat in Turkey. They contribute to a corrective image building and perception, to democratic debates around civil society and political awareness, but also to a space of feelings and emotions of (self-)care and solidarity, not just through their artistic practice but also their presence in the society. The previously established post-migration discourse ^5^ has created opportunities and policy changes in favour of artists with a migration background, particularly Turkish, which has also created a critical space to protect artists of colour from instrumentalization for ideological and conformist purposes.

Nevertheless, the support mechanisms are strained, limited and at risk themselves, particularly after the COVID-19 pandemic. It is of vital importance to map and strengthen the existing support mechanisms in pursuit of helping displaced artists to adapt to the living and working conditions of Germany.

## Methods

The current research is based on a mixed-method approach combining ethnographic research (in-depth interviews) with comparative policy analysis, insights from migration studies and sociology of theatre for context. Semi-structured interviews were conducted between 1 May 2021 and 1 June 2022 with artists and cultural workers from Turkey, residing in Germany, the Netherlands and the UK at the time of the interviews.

Due to considerable risk, all interviews were anonymized with a key as part of the method for processing sensitive qualitative data so that any risk to the individuals resulting from the study will be minimized. Freely given, informed consent is obtained from the participants, by means of a consent form (kept on record) and an information sheet, outlining clearly the study’s key objectives and ethical principles, to guarantee trust and minimize the risks associated with research involving natural, human beings. Extra anonymization in the publication of results was also offered as an option in the consent form.

Secondary data include information available on publicly accessible platforms (websites, social media and online archives giving information on vision statements, application procedures and cultural policy texts). Sources were selected on the basis of the input of the participants in the interviews and a comprehensive literature search on all the relevant support schemes available in Germany to newly arriving artists in (self-)exile and/or at risk. The only exclusion criterion concerned those schemes that do not prioritize those artists.

### Data collection

Interviewees were approached and interviewed both online and face-to-face, following national COVID-19 restrictions. The study totals 30 adult participants of a wide age range, who have come to Germany, the Netherlands and the UK in the last 10 years. The questions asked about the routes and reasons to leave Turkey, the supportive institutions and networks, the sense of new subjectivities in the receiving country or city, the possibilities and limitations of artist production, and the vision of the near or distant future ( Verstraete, 2021a). Upon request, the questions could be shared prior to the interview. The study’s key objectives and ethical principles were provided in written form prior to first contact with and data collection from any participant. The data collection involved only audio recording. Interviews had a duration of on average 50 to 120 minutes.

### Data collection (secondary data)

For the research on the support programs, residencies, institutions and networks, websites as well as flyers of the relevant institutions and organizations were consulted.

### Analysis

Themes such as (self-)care and solidarity *versus* concern were identified from the literature in advance of the data and formed a special focus for the analysis of this study. The context of post-migration discourse was also studied in previous research, prior to the current study. Other themes, such as sustainability and fragmentarization of the existing support programs, were derived from the interviews. The study made use of MaxQDA Plus 2020 software to process policy texts and programs in conjunction with interview transcripts on the basis of tags creating automatic codes to improve and accelerate comparison through the campus license of the Freie Universität Berlin (until September 2023). End user agreements with VERBI GmbH are in place. The study could be replicated with freely available or open-source data analysis alternatives, like QualCoder, Taguette, RQDA, CATMA and Wired-Marker.

### Ethics

Ethical approval was obtained by the Central Ethics Committee of the Freie Universität Berlin (ZEA-Nr. 2020-012). Freely given, written informed consent is obtained from the participants, by means of a consent form (kept on record) and an information sheet (available in English, Turkish and Kurdish), before conducting any interviews, to guarantee trust and minimize the risks associated with research involving natural, human beings.

## Results

### Support programs

Germany’s current policy infrastructure largely splits topics related to Turkey into two separate policy areas that do not interfere with each other: socio-political and developmental issues are treated by the Ministry for International Cooperation as well as the Foreign Offices as a foreign policy topic, whereas labour and visa issues regarding incoming migrants fall under domestic policy by the Ministry of the Interior, the *Bundesamt für Migration und Flüchtlinge* (Federal Office for Migration and Refugees, BAMF).

Besides regulating social insurance for artists and indirect support through the funding of cultural institutions, the Federal Government’s support limits itself to the funding of stays abroad for artists living in Germany. Funded by the Federal Foreign Office of Germany, there is the **Martin Roth-Initiative** (MRI), which is a joint project by IFA ( *Institut für Auslandsbeziehungen*) and the Goethe-Institut, Germany’s cultural institute of the Federal Republic of Germany with a global orientation. MRI supports artists who are committed in their home countries to the freedom of art, democracy and human rights and who are persecuted because of that. They offer temporary residence in Germany or in third countries, but Turkey is not regarded as a high risk. The number of artists they support is very limited and their current orientation is more on Africa and Latin America.

Moreover, the migration policy of Germany is rather intended to manage, control and limit the immigration of foreigners. This makes interconnected issues and local partnerships on a micro-level between the two countries hard to support. On a more macro-level, the European framework does also hardly support individual artists with a special need to leave their countries of origin. Mobility falls largely under the Creative Europe programme and related to that, the European Commission also launched the **S+T+ARTS** (Science, Technology and the Arts) initiative under Horizon2020 to support collaboration between artists, scientists, researchers and engineers, with its own residencies program. Such programs are, however, highly specialized, restricted, competitive, and thus hard to obtain.

As mentioned in the introduction, Germany and Turkey’s societies are interconnected, which binds them in solidarity and politics. There is no support for artists on a state level due to a cultural policy climate that is divided between the federal states. Although the Cultural Foundation of the German Federal State ( ***Kulturstiftung der Länder***) actively supports diversity and the social relevance of art, it is tasked to support institutions (museums and archives), project executing organisations, networks and interest groups on a larger scale. It does offer a limited number of international artist fellowships in combination with residencies, but they are targeting German artists to work abroad (Italy and France). Due to this lack for newcomers, the city has become increasingly a critical actor in this type of support. There are also a few private non-profit support programs that help to strengthen the bilateral dependency and the civil societies within a framework for change.

In Berlin, most central is the fellowship program **‘Weltoffenes Berlin’** by the Senate Department for Culture and Europe ( *Senatsverwaltung für Kultur und Europa*) which was designed to professionally integrate people working in art, media and culture who intend or are being forced to leave their home countries. The grant (a monthly sum of up to EUR 2500) is for a maximum period of a year and is targeted to finance project-related costs to secure independent artistic or creative work in collaboration with a cultural sector stakeholder. This means that the artist who applies needs to be already somehow embedded through their network in the cultural sector in Berlin in order for the application to be successful.

Another Berlin-based fellowship program open to artists has recently been initiated by **Academy in Exile**, under the thematic rubric ‘ Fixing What’s Broken’, from 2021 onwards. Founded in 2017, particularly as a result of the exodus of academics from Turkey, Academy in Exile is a consortium of the Duisburg-Essen University, the Institute for Advanced Study in the Humanities (KWI) in Essen, the Berlin-based Forum Transregionale Studien, and the Free University Berlin. The consortium provides normally fellowships for academics that are threatened or delegitimized in their home countries. Yet it now offers also 3 three-month residency fellowships in Berlin (a monthly stipend of EUR 2500 plus travel expenses), funded by the Allianz Kulturstiftung, to artists and cultural producers who advocated human rights, democracy, free expression and/or who have been displaced because of their work.

More Germany-wide, the non-profit, originally French organization, **Aid A – Aid for Artists in Exile** (previously AIDA, founded in 1979), supports artists around the world who have been threatened in their home countries particularly for defending artistic freedom, democracy, and human rights and who are, therefore, prevented from continuing their work. Similarly, there is since 2013 the non-profit network, **Artists at Risk (AR)**, that recently also partnered with Germany’s cultural institute of the Federal Republic of Germany, the Goethe-Institut to coordinate a German network of hosting institutions that support persecuted art practitioners (like Kurdish artists Barış Seyitvan and Güllü Özalp), facilitating their safe passage from their countries of origin, hosting them at residencies, and curating related projects. Since the demand is high, the residencies scattered over Europe and the priorities of who to support change more quickly depending on global dynamics, such non-profit organizations and networks cannot offer sustainable solutions to many artists for a longer period of time, yet they do create awareness, constantly map the need and provide networks of care and solidarity. One such network that contributes to awareness is Kopuntu, a solidarity network focusing on the new generation diaspora.

As a private initative since 2000 funded by the Allianz insurance group, the Allianz Kulturstiftung collaborates actively with the European framework to support contemporary art, new music, literature and translation, and European development. It supports a residency program with annual stipends for four up to eight months (up to EUR 2500 per month) to foster exchange at the **Tarabya Cultural Academy** in Istanbul, either for individuals from Germany or for tandem-partners with one partner from Germany, one based in Turkey. This partnership favours ‘exceptionally qualified artists and cultural professionals’ and is more targeted towards exchange than support for artists who are in real danger. For the latter, the Allianz Kulturstiftung has a very small program since 2018, **‘ Torschreiber am Pariser Platz’**, which only supports one writer in exile per year up to six months. Since 2020, Esra Kücük assumed the role as Chief Executive Officer. She previously worked at the Gorki Theater in the GORKI FORUMS particularly on the topic of exile. This may be hopeful for the widening of the Artist

For threatened authors, academics and journalists, there is also **PEN International’s Writers in Exile** program with offices in Istanbul ( PEN Turkey Centre) and Darmstadt ( German Centre). The latter’s support is based on the priority and severity of the risk, which they also constantly monitor (in an annual case list). PEN International is an organisation funded by donations and different partners; the German branch is supported by the Minister of State for Culture and Media at the Bundesregierung.

While most support programs and residencies offer immediate financial relief for the early days of the relocation and some even help to embed artists at risk also in the civic, cultural and social ecologies of the new home, they are limited and not sustainable in the long run. Most newcomers do not speak German and adapting to the new living situation, sometimes with a family, in a new cultural and linguistic environment requires much more time and energy than that the support program can help with. Artists who left Turkey are also at different risk levels, which often excludes them from the support programs. Some lack the support networks for getting institutional support, others are more focused on educating themselves further or coming temporarily for exchange, which complicates a longer stay. They are also generally different to economic migrants as their mobility was often not as planned or coordinated towards a job.

For a non-exhaustive list of supportive institutions and organizations in Germany together with their modes of support, please see Table 1.

**Table 1.** Non-exhaustive taxonomy of German institutions who supported artists at risk ^6^.

ORGANISATION	DESCRIPTION	TYPE OF SUPPORT
Akademie der Kunste (AdK) (Berlin)	One of the oldest cultural institutions in Europe (since 1696) which offers an exhibition and event location for concerts, readings, award ceremonies, dance/theatre performances, film screenings and public debates on art, cultural heritage and cultural policy as well as one of Europe’s most important interdisciplinary archives with its own publication series (Journal der Künste, catalogues and books).	Supported by the Minister of State for Culture and Media and Neu Start Kultur (emergency fund for culture and media from the Bundesregierung), AdK supports a wide range of artists though not with a special category for artists at risk, by means of: • international scholarship programmes, including an emergency fund for young artists (based on donations) • the JUNGE AKADEMIE programme shaped by each year's group of fellows together with mentors and members of the Akademie, which has supported artists from Turkey to organize public debates on current issues related to art production in Turkey • an online petition in favour of artistic freedom initiated by the European Alliance of Academies (founded in 2020 to speak out for freedom of art in Europe) together with the European Center for Constitutional and Human Rights (ECCHR)
Apartment Projekt Berlin (Berlin)	Under the direction and supervision of Selda Asal, Apartment Project is since 2012 an exhibition non-profit art space in Berlin (modelled after its Turkish counterpart, Apartman Projesi since 1999 in Istanbul) for collaborative artistic practice with a focus on global-local idea exchange, particularly with an interest in politically engaged art from Turkey, the South Caucasus, Iran and the Balkans.	Ongoing support of mostly visual artists but also critical performance artists and filmmakers by means of • thematic programs for artist talks, including on exile and on precarity in Chromatic Wednesdays • support of projects through an exhibition and performance space with social media advertising • the project “ His TV” as a tv station by artists who have recently moved from Turkey to Germany • the collaborative project “ Condition Room” to give voice to artists from Turkey reflecting on the complicated situation back home
bi’bak (Berlin)	Under direction of Can Sungu and Malve Lippmann, bi’bak is since 2014 a non-profit organization and project space in Berlin that focuses on transnational narratives, migration, global mobility and their aesthetic dimensions. It has currently a special thematic focus on repressive regimes.	Ongoing support (from diverse supporters including the Berlin Senate for Culture and Europe) for new wave artists, filmmakers and curators in favour of the LGBTQ+ community and equal participation, by means of • curated thematic film programs • expert talks between artists, filmmakers and academics • collaborative projects support and workshops • research-based exhibitions • critical publications
Fringe Ensemble (Bonn)	Under direction of founder Frank Heuel (since 1999), this theatre ensemble with its homebase in the Theater im Ballsaal in Bonn has coproduced productions and projects in Istanbul, including Map to Utopia (2019–2022).	Supported by the city of Bonn, the Ministry of Culture and Science for the State Nordrhein-Westfalen and several other regional and state cultural funds, the fringe ensemble/Türkei GbR has supported Kurdish artist Mîrza Metin from Istanbul between 2017 and 2020 on project basis in Bonn and helped to tour his productions in coproduction with Şermola Perfomans, Istanbul through Germany and Turkey.
Hafiza Merkezi Berlin (Berlin)	Originally a humanitarian organisation since 2011 in Istanbul which opened since 2018 an independent non-profit organization in Berlin recognized as charitable, under supervision of Gamze Hızlı, Murat Çelikkan, Özlem Kaya, to promote human rights through international advocacy and solidarity actions with a comparative perspective to tackle the global backlash of rising authoritarian governments, increasing nationalism and right-wing extremist movements.	Supported by the Federal Foreign Office, Stiftung Mercator, Civil Rights Defenders, and Human Rights organizations globally. It wants to be a hub that facilitates exchange between civil society actors of Turkey and Germany/Europe, by means of • project-based support for a ‘European-Turkish Network for Democracy and Civil Society’, ‘Defending Others, Liberating Themselves: WHRD’s Experiences in Turkey, ‘In Good Times and Bad: Living Together’ • critical publications of reports on social and political matters from a comparative perspective to Turkey
Maxim Gorki Theater (Berlin)	Under direction of Şermin Langhoff since 2012, the Gorki Theater is one of Berlin’s big stages for theatre professionals from its own ensembles (including Exil Ensemble) and also sometimes from Turkey (like German- born Turkish actor Barış Atay) with a strong connection to current social debates, including questions of postmigration, identity, belonging and the construction of nation.	Ongoing support of artists at risk in terms of shelter, project application support and professional means to continue working by means of • curation of an exhibit-parcours in the Berlin *Herbstsalon* (Berlin Autumn Salon, especially nr.5) • institutional support through the Young Curators Academy • regular column of exiled journalist Can Dündar as well his "Museum of Small Things" • exhibition of exiled Kurdish artist/journalist Zehra Doğan (Prison No.5) and Timur Çelik (further supported by Künstlerhaus Bethanien) • support for Osman Kavala’s release
Nexus (Bonn)	Under direction of founder Mîrza Metin (since 2018) in collaboration with Frank Heuel (fringe ensemble, Bonn), this Kurdish-German theatre network offers a platform to Kurdish theatre artists (19 official members).	Supported by the Ministry of Culture and Science for the State Nordrhein- Westfalen, this talent network offers logistic and communicative support to Kurdish artists in Germany, by means of • facilitating workshops • supporting existing projects with the fringe ensemble • supporting the development of an archive on Kurdish theatre artist living in Germany by Mîrza Metin and Hicran Demir
Oyoun (Berlin)	Co-founded in 2020 by Louna Sbou and other members of the non- profit Kultur NeuDenken gUG (limited liability), this non-profit cultural centre which helps to initiate, conceptualize and realize artistic and socio-cultural projects with migrant, decolonial, queer*feminist, neurodiverse and class-critical perspectives in Berlin and internationally (with intersectionality as a key guiding principle).	Supported by Senate Department for Culture and Europe, this newly acquired cultural space and non-profit enterprise company support new wave artists with a migration and queer background by means of • logistic support for collaborative experiment, discussions, rehearsal and production by offering spaces and social media promotion through their website, Facebook page and newsletter • online discussion on topical issues through contributions on their website • fellows in residency (one at the moment, Mustafa Elsiddig from South Sudan)
Theater28/Tiyatro28 (Berlin)	Founded in 2010 by Ufuk Güldü, the theatre focuses on the local Turkish community of Berlin’s Soldiner Quarter as an intercultural theatre and cultural/social meeting centre by means of theatre pedagogy projects (children's theatre), theatre and film projects on social issues.	Supported by the community and diverse institutions and partners (also in Turkey), Theater28 has presented new plays in Turkish, both from Turkey and from Turkish artists in Berlin. The exchange with Turkey concentrates in the annual German-Turkish Theatre Days (Deutsch-Türkische Theatertage) since 2016 with productions from both state/city theatres and independent theatres from Turkey.

### Common deficiencies

Despite the many programs, residencies, networks and institutions that have supported newly immigrating, often vulnerable artists who have left Turkey for diverse reasons, in-depth interviews show that artists still face many problems.

1. A most common problem is that the existing support is temporary, mostly focusing on project-based labour and thus output-oriented, meritocratic, and limited in responding to all the specific needs of the artist who is trying to find their way in the new living situation, the associated bureaucracy as well as the art industry and job market in Germany. The Maxim Gorki Theater is overstrained with the demand for support, and often it takes more time for artists to be redirected to other opportunities as the information is scattered. Support programs and residencies also usually do not offer space and time for learning the language and culture (which takes at least one additional year beside any art projects). Neither do such means of support focusing on carrying on jobs always help to solve any psychological effects of the loss of identity and the looming financial (and in some cases, legal) precarity in a context of living in ongoing short-term protection. Some carry on by moving from one support system to another, with periods of severe precarity, others are resourceful in producing on their own with partial support of coproductions. But there is always a sense that the support will come to an end one day, as public and political interest for the silent exodus from Turkey is waning and other exiles and refugees from other countries are arriving.
2. A second problem is the disparity between the generations of Turkish immigrants and citizens with a Turkish migration background in Germany. Historically and legally, the new wave benefits from the presence of the older generations, but they do not necessarily stand on their shoulders. In spirit and perhaps ideology, they have closest affinity with leftist artists, intellectuals and journalists who fled Turkey after the 1980 military coup and its subsequent conflicts in the Kurdish region in the 1990s, yet there is a great legal difference as a big portion of these migrants in the 1980s arrived as political refugees and asylum seekers, whereas the new wave after 2013 has been arriving with more privileges. The latter are also defined in the press as Turkey’s ‘brain drain’ ( *BBC News*, 28 Dec. 2017) or ‘loss of intellectual elite’ ( *Die Welt*, 7 Feb. 2019), which has created caution in the older generations, particularly the guest worker generation and those voting conservatively. The generational disparity in worldview and political perspective on Turkey limits the solidarity inside the Turkish speaking communities, which by themselves have been struggling to muster support for their own cultural centres and art distribution channels (like the youth association DIDF-Jugend, the previous migration centre Allmende, or the theatre for the first-generation Turkish migrants, Tiyatrom in Berlin).
3. The public debates on special support for migrant artists or those with a migration background in the post-migrant scene have also created a clear break from any support mechanisms for *Sozialarbeit* (social work) that would undermine the artistic qualities and opportunities to professionally exchange know-how. This is an acquired step forward, no doubt, yet it complicates the support of vulnerable artists under the German Act on Equal Treatment as their precarious labour situation should also be seen as part of anti-discrimination laws and support for excluded groups. This creates new inequalities in a scene that has been already overburdened in its struggle against institutionalized inequality, only highlighting more the generational disadvantage that newcomers suffer from.

The specific treatment of artists in exile and/or at risk necessitates also the widening of the public debate and support as they take part of larger waves of ongoing migration affecting diversity issues in society. Most commonly, artists at risk actively engage in awareness and support for other exilic groups in Europe (like currently, the Ukrainians) and the complexity of precarity. Their precarity (whether it be financial, legal, institutional, geographic or cultural) is part of their new identity formation, and their specific stories metonymically help to uncover bigger issues in our modern culture and society. Yet the necessary widening of the scope of support complicates the specificity of the needs of every newly arriving community, and it adds extra risk and vulnerability to those who are trying to find sustainable means and solutions to stay in Germany.

## Conclusion

### Improving existing support: Harnessing networks, fostering (self-)care

1. A first point for improvement concerns the current fragmentation of the existing support mechanisms. Though in the early days after the attempted coup in Turkey, there seemed political good will to strategize the support for the incoming flow of artists from Turkey, just like the academics and journalists, a strategy document that would coordinate targeted themes and existing support structures which could be implemented by theatres and cultural institutions has been lacking. The result is that many support programs work on an ad-hoc basis depending on priorities and risk levels that are often politically defined and motivated, as well as very differentiated from one to another. In order to respond to more sustainable needs of very diversified communities, targeted support could benefit from **a more coordinated effort** by building relationships and communication channels with the specialized networks that enhance diversity and awareness regarding migrants, like the International Cities of Refuge Network ( ICORN). Information and collaboration are key.
2. A second point concerns the **need for representation**. On the one hand, as the postmigrant theatre scene has struggled for, it is vital to have representatives of exiled artists at risk on decision making boards. On the other hand, the theatre stage as well as the use of digital technologies have the ability to empower voices, strengthen identities and enhance visibility for migrants’ precarity and political concerns ( Nedelcu & Soysüren, 2022). Many support programs are directly targeted towards the means of production to reclaim such spaces for representation, yet as a horizon of expectation or even requirement, this is also seen as a problem: artists may feel the need to have to address migration issues and/or exploit their newly ascribed identity in order to get opportunities, thus compromising their artistic autonomy. Indeed, the role of theatre in issues of identity, has proven that it can shift the debate. Yet, as discourses of postmigration have proven, we must be careful with treating artists as ambassadors of a culture or as an ideological tool to contest certain symbolic politics, whether it be domestically or abroad.
3. A third, most prevailing proposition to improve the life situation of the artist at risk is to **strengthen** currently forming artist **networks** **to enhance communities in self-care**. Recent debates around the social engagement of theatre practice, particularly in COVID-times, have shifted towards topics of attentiveness, ethics, relationality and responsiveness in a larger framework of (self-)care. The latter should not only be understood in entrepreneurial terms but also broader, in the sustainable support of personal growth by means of listening, understanding, responding, and potentially rethinking our attitudes ( Stuart & Thompson, 2020). Care shares similarities with ‘concern’ (both derived from Latin ‘cura’) which is the driving force of most support mechanisms, yet it is also different in that ‘care’ has stronger ethical, material and affective connotations. According to de la Bellacasa, it “extends a vision of care as an ethically and politically charged *practice*” ( 2011: 90). The latest efforts by the Fonds Darstellende Künste (fonds-daku) during the COVID19-pandemic to support artists through their support programs #TakeCare and #TakeHeart, in collaboration with NEUSTART KULTUR, are promising in this regard as they are more focused on research, development, and restart of cultural life after the pandemic than on output. Yet, besides a call for artists from Ukraine in their special #TakeAStand residence program ^7^, artists at risk do not generally benefit from these funds.

Through Germany’s support systems, we should avoid alienating or traumatizing those who have fled a politically volatile situation in their home country twice over, not by giving them preferential treatment as rules of exception, but by fully including them as equal partners in the receiving society, its cultural industry and job markets, as a requirement to fulfill this inclusive vision of relational (self)care in the future. Special programs are important to target specific needs in situations of risk, yet a two-tier support system that separates artists in terms of privilege or origin should be prevented.

### Policy recommendations

Based on a mapping of support mechanisms as well as interviews concerning artists at risk from Turkey in Germany and Europe, the report proposes the following recommendations:

1. a more integrated, strategic approach that combines better coordination, collaboration and information exchange involving the different actors, institutions, networks, support services and residencies of individual theatres and cultural centres;
2. more coordinated support in helping artists and cultural professionals from Turkey to entering the German/European cultural market;
3. affirmative action in favour of inclusivity, diversity and representation of artists at risk, including participation of representatives of disadvantaged groups in boards and commissions;
4. a shift in focus from immediate concern to more sustainable (self-)care in the scaffolding of support programs and strengthening of existing networks, in favour of an inclusive society.

### Ethics and consent

Ethical approval was obtained by the Central Ethics Committee of the Freie Universität Berlin (ZEA-Nr. 2020-012). Freely given, written informed consent is obtained from the participants, by means of a consent form (kept on record) and an information sheet ( Verstraete, 2021b; Verstraete, 2021c; Verstraete, 2021d), before conducting any interviews, to guarantee trust and minimize the risks associated with research involving natural, human beings.

## Data Availability

Processed data are shared through the project website ( http://www.exiledlives.eu), including video recordings of events and podcast episodes for which freely given, informed consent is obtained from all participants. On the website, there is detailed information about the project’s aims as well on where the findings of the project will be disseminated. This includes publications, conference presentations, public lectures, round tables, podcast appearances and other forms of communication and dissemination. The data underlying the results cannot be shared for confidentiality reasons. For this particular publication, any additional information on data that is not yet retrievable online could be requested by contacting the researcher by E-Mail: info@exiledlives.eu, by stating the purposes of their request. Due to the strict confidentiality offered to the interlocutors, no datasets of interviews will be shared without extra consent by them. Zenodo: Exiled Lives on the Stage: Questions for Interviews with Artists in (Self-)Exile and/or at Risk from Turkey. https://doi.org/10.5281/zenodo.7950715 ( Verstraete, 2021a). This project contains the following extended data: ExiLives Questions-Pieter Verstraete.pdf (The questions of the study) Zenodo: ExiLives: Participant Information Sheet. https://doi.org/10.5281/zenodo.7950787 ( Verstraete, 2021b). This project contains the following extended data: exilives infosheet easy english.pdf (Participant information sheet in English) Zenodo: ExiLives: Katılımcı bilgi formu. https://doi.org/10.5281/zenodo.7950808 ( Verstraete, 2021c). This project contains the following extended data: exilives infosheet easy turkish.pdf (Participant information sheet in Turkish) Zenodo: ExiLives: Kaxêza agahdariyên bo beş daran. https://doi.org/10.5281/zenodo.7950801 ( Verstraete, 2021d). This project contains the following extended data: exilives infosheet easy kurdish.pdf (Participant information sheet in Kurdish) Data are available under the terms of the Creative Commons Attribution 4.0 International license (CC-BY 4.0).

## Footnotes

^1^ The present policy brief will refer to ‘Turkey’ in English despite governmental attempts to rename it. In December 2021, the Turkish government namely announced a ‘national rebrand’ that dispenses with its 100-year-old Anglicized name due to its association with the same-named bird, and demanded to replace it by its Turkish name, 'Türkiye’. This was confirmed on 1 June 2022 at the United Nations with a request in a letter from Foreign Minister Mevlüt Çavuşoğlu ( Shankar & Timur, *The New York Times*, 2 June 2022). Since *Türkiye* has not become the standard yet and language develops different than governments may want, I will stick to *Turkey*.

^2^ The term ”new wave“ is literally used in two subsequent FaceBook groups where newcomers from Turkey refer to themselves as “New Wave Berlin” (the current one was created on 1 October 2020).

^3^ The latest role that Turkey played as peace negotiator between Russia and Ukraine for the UN Security Council in March 2022 but also for the organization of a peace summit together with the UN and Qatar on behalf of Afghanistan confirm that image in international affairs.

^4^ I would like to acknowledge that the current policy brief is inspired by the aims and format of Telli-Aydemir and Cagla’s policy brief.

^5^ The postmigrant discourse was set in motion by previous Ballhaus and now Gorki intendant Shermin Langhoff alongside author Feridun Zaimoğlu and other activists and cultural practitioners, whose usage of the term was borrowed from an anglophonic academic context. The term was, for instance, used in an academic essay collection by Baumann and Sunier, entitled *Post-Migrant Ethnicity: De-essentializing Cohesion, Commitments, and Comparison* ( 1995). The concept has been developed in Germany primarily through artist-led activities and discussions between 2004 and 2008, especially around the Ballhaus Naunynstrasse, whose success has influenced academic reception and policymaking to the point that it has become mainstream. See: Petersen, Anne Ring, Moritz Schramm and Frauke Wiegand, “Postmigration as a Concept (Reception, Histories, Criticism)”, in Moritz Schramm, Sten Pultz Moslund, Anne Ring Petersen *et al.* (eds.), *Reframing Migration, Diversity and the Arts: The Postmigrant Condition*, New York/London: Routledge: 1-64.

^6^ This list is not exhaustive and focuses mostly on Berlin’s dense ecology of institutions and those that are most visible in their support of incoming artists from Turkey. Other institutions, like Radialsystem, Hebbel am Ufer (HAU), Schaubühne, Heimathafen Neuköln, Neuköllner Oper, Haus der Kulturen der Welt (HKW) and Humboldt Forum in Berlin as well as the Münchner Kammerspiele in Munich have a history of project-based collaborations (some even directly on exile) and sometimes residencies for artists at risk too. For the fine arts, the Neuer Berliner Kunstverein (n.b.k.) and B.L.O.-Ateliers (through its “ Hier&Jetzt: Connections” residency program) in Berlin have also supported visual artists from Turkey.

^7^ See the overview by the Bundesverband Darstellende Künste about the support programs for Ukrainian artist refugees.
