# Supplementary material for: Exiled lives on the stage: Support networks and programs for artists at risk from Turkey in Germany
Source: Open Res Eur. 2023 Jul 6;3:109. [Version 1] doi: 10.12688/openreseurope.15726.1 (PMC10457555; doi:10.12688/openreseurope.15726.1)
Supplement: Peer review history

# Peer review: Reviewer response for version 1

**DOI:** 10.21956/openreseurope.16990.r33607

**Published:** 2023-08-25

**Authors:** Kerem Halil-Latif Oktem

**Affiliations:** 1 Ca' Foscari University of Venice, Venice, Veneto, Italy

This paper gives a concise and well-researched synopsis of the care and self-care mechanisms available for artists at risk from Turkey. There is an insightful discussion of the different networks, initiatives and institutions, their activities, and the limitations of their work. As this is not an academic article *strict sensu *with a strong theoretical grounding, but a policy-oriented paper, questions about the originality of the argument are not relevant. Overall, the report leaves the reader much better informed about the problems and the possible solutions of artists at risk from Turkey. 

 My limited criticism would aim at the policy recommendations which struck me as slightly too general. I believe that these recommendations would be stronger, if they included concrete suggestions for the trajectories and choices for existing actors, networks, and programs that address the durability of such programs. Alternatively, the author could de-emphasize the policy recommendations and conclude on a more theoretically grounded note, reflecting on the necessity and sensibility of such ad-hoc support programs (also including case studies from other countries, if they do exist) and their efficacy (i.e. how long can such programs be continued without turning ‘artists at risk’ into ‘artists dependent on social welfare’).

If any, are all the source data and materials underlying the results available?

Yes

Is the work original in terms of material and argument?

Yes

Is the work clearly and cogently presented?

Yes

Is the argument persuasive and supported by evidence?

Yes

Does the research article contribute to the cultural, historical, social understanding of the field?

Yes

Does it sufficiently engage with relevant methodologies and secondary literature on the topic?

Yes

Reviewer Expertise:

Turkish Studies, Politics, Autocratization and democratisation

I confirm that I have read this submission and believe that I have an appropriate level of expertise to confirm that it is of an acceptable scientific standard.

# Peer review: Reviewer response for version 1

**DOI:** 10.21956/openreseurope.16990.r33608

**Published:** 2023-08-18

**Authors:** Deniz Basar

**Affiliations:** 1 Bogazici University, Istanbul, Turkey

This article fills a very important gap in the field of research centered on the new wave of intellectual immigration from Turkey that took place in the 2010s. In particular, the fact that this research does not focus on the experiences of one or a few individuals, but rather attempts to capture a sense of the field that the immigrant artist from Turkey in particular is trying to integrate by analyzing and comparing in-depth interviews with policy reports, marks the research as a unique contribution to the field.

 It is also interesting and innovative to document the inter-community tensions between Turkish-speaking groups in Germany, and the class and education-based differences between earlier generations of immigrants and the post-2010s immigrants.

 The notes I mention below do not need to be integrated into the current form of the article, since this peer-review document is currently paired with the article. Please consider them as notes for readers and future researchers. 

 There are two points I would like to note about this research that I think can be improved, both of which are about adding some important nuances to the background of the phenomenon being researched here. The first is to perhaps briefly mention the cancellation of the peace negotiations between the Turkish state and the Kurdish movement that took place between 2012 and 2015, since this political rupture also affected the aftermath of the 2016 military coup attempt, which significantly contributed to the wave of dissident intellectuals who were forced to leave Turkey. In order to strengthen the article on this front (and to show the relationship between the end of the peace negotiations and its aftermath, on the intellectual exodus from Turkey), I can recommend briefly quoting this article at a relevant point:

- Özgül Akinci (2018) Performing Academia in Public Space in Turkey, Performance Research, 23:2, 44-48, DOI: 10.1080/13528165.2018.1464753

The second point that I think could be improved is a brief overview of the history of Turkish migrant theater in Germany and the reasons for its discontinuity up until now by citing some sources on the topic. Two sources that immediately come to mind are:

- Ela E. Gezen. Brecht, Turkish Theater, and Turkish-German Literature: Reception, Adaptation, and Innovation after 1960. Rochester, NY: Camden House, 2018.
- Erol M. Boran. Die Geschichte des türkisch-deutschen Theaters und Kabaretts - Vier Jahrzehnte Migrantenbühne in der Bundesrepublik (1961-2004). Transcript-verlag, 2022. (This work was also available in the form of Boran's dissertation at the time of Verstraete's research.)

In addition, a minor point is that on page 7 of the pdf version of the document, the paragraph that begins with “As a private initative since 2000 funded by the Allianz insurance group” ends with an unfinished sentence that cuts off the sentence as follows: "This may be hopeful for the widening of the Artist”.

 Despite these two brief points, which are preliminary information to better ground the research in its two contexts (in Turkey and in Germany), I think the research is a very important contribution to the literature on the intellectual exodus from Turkey in the 2010s.

If any, are all the source data and materials underlying the results available?

Partly

Is the work original in terms of material and argument?

Yes

Is the work clearly and cogently presented?

Yes

Is the argument persuasive and supported by evidence?

Yes

Does the research article contribute to the cultural, historical, social understanding of the field?

Yes

Does it sufficiently engage with relevant methodologies and secondary literature on the topic?

Yes

Reviewer Expertise:

Turkish contemporary theatre, performative politics, public sphere studies, puppetry

I confirm that I have read this submission and believe that I have an appropriate level of expertise to confirm that it is of an acceptable scientific standard.

## References

[1] : Performing Academia in Public Space in Turkey. *Performance Research* .2018;23(2) : 10.1080/13528165.2018.1464753 44-48 10.1080/13528165.2018.1464753

[2] : Brecht, Turkish Theater, and Turkish-German Literature: Reception, Adaptation, and Innovation after 1960. *Rochester, NY: Camden House* .2018;

[3] : Die Geschichte des türkisch-deutschen Theaters und Kabaretts - Vier Jahrzehnte Migrantenbühne in der Bundesrepublik (1961-2004). *Transcript-verlag* .2022;

# Peer review: Reviewer response for version 1

**DOI:** 10.21956/openreseurope.16990.r33609

**Published:** 2023-08-18

**Authors:** Ozlem Savas

**Affiliations:** 1 Comparative Cultural and Social Anthropology, European University Viadrina, Frankfurt an der Oder, Brandenburg, Germany

The research article “Exiled lives on the stage: Support networks and programs for artists at risk from Turkey in Germany” critically explores the institutions, networks, and programs that aim to support displaced artists and to facilitate their integration into the artistic labor market, based on a study of the case of artists who recently left Turkey mainly due to political repression and settled in Germany.  As well as extensively mapping the existing support systems, the article uncovers their deficiencies that put artists in precarious positions and negatively affect their artistic careers and individual well-being. Furthermore, the author offers valuable suggestions to improve the functioning and the sustainability of the support systems. Such a constructive critical approach to the support mechanisms is vital in the wake of growing number of artists and academics who are fleeing their countries due to political oppression, turmoil and violence, as well as flourishing support programs that face some challenges. This study delves into significant questions and represents great potential to contribute to the policies of support systems.  

   

 Yet, the article lacks theoretical and conceptual framework since it does not engage with the existing literature sufficiently. For this reason, the work serves more as a policy paper or a project report than a research article in its current form and cannot realize its full potential to contribute to the scholarly understanding of the field. In the analysis section, it is mentioned that the themes of (self-)care and solidarity and the context of post-migration discourse were previously studied through the relevant literature. I would suggest the author to theoretically engage with these concepts and debates in this article too. The short discussion of (self-)care vs. concern in the conclusion could be expanded and moved to the beginning of the article. What does self-care mean in “entrepreneurial terms”? How do the forms of care suggested in this study differ from neoliberal discourses and industries of self-care? I think that the article can substantially contribute to the growing scholarly interest on care practices and discourses, if the project findings are discussed through the theoretical frameworks. I would also be interested in reading more about the support programs #TakeCare and #TakeHeart. In addition, the article often mentions “post-migration discourse” but does not discuss what it is. A theoretical discussion of the concepts of postmigration and postmigrant society could support the article’s arguments. Lastly, I would recommend a brief discussion of the literature on the topics of precarity in migration contexts and the general precarity in artistic labor markets.

 The argument on the generational disparity among people who migrated from Turkey at different times and its impact on the possibilities of solidarity is quite intriguing. I was wondering if the author could further elaborate on this. The argument could be enhanced by describing the disparity in worldview and political perspective in more detail and discussing the relations of newcomers with the cultural centers established by older generations. For example, are there some forms of solidarity and collaboration between different generations in the art scene?   

 Since in-depth interviews have been employed to detect deficiencies of supports systems, I would be interested in reading more input from the interviews conducted. Also, since the methodology has been described as ethnographic research, I was wondering if there has been participant observation involved in the field study. Some input from the artistic and cultural events in which the author participated, if any, could enhance the overall article.  

 The introduction presents a well-written review of the recent political situation in Turkey that causes the exodus of artists, as well as the historical connections between Germany and Turkey that have been shaped by multiple migrations. However, it does not explain aims, scope and the conceptual framework of the article. These could be addressed in the introduction and the detailed discussion of the background of displacement from Turkey can be possibly made into a separate section. In addition, recommendations to improve the support systems that are discussed in the conclusion can be made into a separate section. And, the conclusion can rather restate and wrap the main findings and arguments of the study.

If any, are all the source data and materials underlying the results available?

Yes

Is the work original in terms of material and argument?

Yes

Is the work clearly and cogently presented?

Yes

Is the argument persuasive and supported by evidence?

Yes

Does the research article contribute to the cultural, historical, social understanding of the field?

Yes

Does it sufficiently engage with relevant methodologies and secondary literature on the topic?

Partly

Reviewer Expertise:

Migration studies, media studies and ethnography; research expertise on the recent displacement from Turkey.

I confirm that I have read this submission and believe that I have an appropriate level of expertise to confirm that it is of an acceptable scientific standard, however I have significant reservations, as outlined above.
